# Dysphagia symptoms in obstructive sleep apnea: prevalence and clinical correlates

**DOI:** 10.1186/s12931-021-01702-2

**Published:** 2021-04-21

**Authors:** Nicole Pizzorni, Dejan Radovanovic, Marica Pecis, Rosaria Lorusso, Federica Annoni, Alice Bartorelli, Maurizio Rizzi, Antonio Schindler, Pierachille Santus

**Affiliations:** 1 Phoniatric Unit, Ospedale L. Sacco, ASST-Fatebenefratelli-Sacco, Department of Biomedical and Clinical Sciences “Luigi Sacco”, Università degli Studi di Milano, Via GB Grassi 74, 20154 Milan, Italy; 2 Division of Respiratory Diseases, Ospedale L. Sacco, ASST-Fatebenefratelli-Sacco, Department of Biomedical and Clinical Sciences “Luigi Sacco”, Università degli Studi di Milano, Milan, Italy

**Keywords:** Obstructive sleep apnea, Deglutition disorders, Polysomnography

## Abstract

**Background:**

Epidemiology of dysphagia and its drivers in obstructive sleep apnea (OSA) are poorly understood. The study aims to investigate the prevalence of dysphagia symptoms and their association with demographic and clinical factors in patients with OSA.

**Methods:**

Patients with OSA referring to an Academic Sleep Outpatient Clinic were enrolled in a prospective study. Demographic, clinical characteristics, and OSA symptoms were collected. All patients underwent home sleep cardiorespiratory polygraphy and the Eating-Assessment Tool questionnaire (EAT-10) to investigate dysphagia symptoms. Patients with a positive EAT-10 were offered to undergo a fiberoptic endoscopic evaluation of swallowing (FEES) to confirm the presence of dysphagia. FEES findings were compared with a healthy control group. Univariate and multivariate analyses were performed to assess predictors of dysphagia.

**Results:**

951 patients with OSA (70% males, age 62 IQR51-71) completed the EAT-10, and 141 (15%) reported symptoms of dysphagia. Female gender (OR = 2.31), excessive daily sleepiness (OR = 2.24), number of OSA symptoms (OR = 1.25), anxiety/depression (OR = 1.89), and symptoms of gastroesophageal reflux (OR = 2.75) were significantly (p < 0.05) associated with dysphagia symptoms. Dysphagia was confirmed in 34 out of 35 symptomatic patients that accepted to undergo FEES. Patients with OSA exhibited lower bolus location at swallow onset, greater pharyngeal residue, and higher frequency and severity of penetration and aspiration events than healthy subjects (p < 0.05).

**Conclusion:**

A consistent number of patients with OSA show symptoms of dysphagia, which are increased in females and patients with a greater OSA symptomatology, anxiety and depression, and gastroesophageal reflux. The EAT-10 appears a useful tool to guide the selection of patients at high risk of dysphagia. In clinical practice, the integration of screening for dysphagia in patients with OSA appears advisable.

**Supplementary Information:**

The online version contains supplementary material available at 10.1186/s12931-021-01702-2.

## Background

Swallowing is a highly complex sensorimotor process requiring adequate neuromuscular coordination, strength, precision, timing, speed, and motor planning [1]. Any alteration to these components may lead to oropharyngeal dysphagia, i.e. an impaired bolus transit from the mouth to the esophagus. Dysphagia is associated with severe complications, such as aspiration pneumonia, malnutrition, and dehydration, that impact survival, clinical management, and health costs [2].

Dysphagia may occur in patients with obstructive sleep apnea (OSA). Although the pathophysiology of dysphagia in OSA is not clearly understood, the literature suggests that dysphagia in OSA may be the result of sensory and motor changes of the pharynx and altered swallowing-breathing integration [3–5]. Data on the epidemiology of dysphagia in OSA are scarce. Based on a recent systematic review, studies instrumentally assessing dysphagia in patients with OSA reported a prevalence of 20–77%. However, the number of studies was scarce (n = 8), they were based on small samples (< 75 patients), not selected based on dysphagia symtpoms [6]. Therefore, no data exists on dysphagia characteristics in OSA patients who complain of dysphagia. One study from Japan analyzed dysphagia symptoms in a large sample of 507 patients with OSA and found a prevalence of 16% [7]. However, the study used a non-validated questionnaire and no confirmation of dysphagia by instrumental assessment was conducted. The review also reported contradictory findings on which factors (such as age, gender, OSA severity) are associated with dysphagia and no study analyzed the interaction of multiple factors on dysphagia development in OSA [6]. Knowledge of risk factors for dysphagia in OSA would assist clinicians in the identification of patients requiring a comprehensive swallowing assessment.

The study aimed to: (i) investigate the prevalence of dysphagia symptoms in patients with OSA, (ii) analyze the association between dysphagia symptoms and demographic and clinical variables, and (iii) describe objective signs of dysphagia in symptomatic patients with OSA. It was hypothesized that: (i) the prevalence of dysphagia symptoms would be similar to that reported by the Japanese study [7], (ii) dysphagia symptoms would be associated with age and the complexity of the clinical condition, and (iii) symptomatic patients with OSA would exhibit objective signs of impaired swallowing.

## Material and methods

A prospective single-center study was conducted on outpatients referring to the Sleep Clinic of the Luigi Sacco University Hospital in Milan that underwent a home sleep cardiorespiratory polygraphy for suspected OSA. Patients were consecutively enrolled from April 2018 to November 2019. The study was approved by the Luigi Sacco Hospital ethic committee (P.12042021) and was conducted following the principles of the amended Declaration of Helsinki (2013). Written informed consent was obtained from all patients.

### Patients

Adult patients (age ≥ 18) with a confirmed diagnosis of OSA and preserved reading skills were included in the study. Exclusion criteria were: other concomitant chronic respiratory diseases such as asthma or chronic obstructive pulmonary disease; known oesophageal diseases (Barret’s esophagus, esophageal cancer, gastric resection, esophageal achalasia), known neurological diseases associated with dysphagia (stroke, brain injury, neurodegenerative diseases, muscular dystrophy, myasthenia gravis), head and neck cancer, presence of central apneas, pregnancy, and drug abuse.

Demographic, polysomnographic, and clinical data were collected. Patients were asked to complete the Italian version of the Eating-Assessment tool (EAT-10) [8, 9], a self-administered questionnaire for the screening of dysphagia. Based on previous validation, patients who scored ≥ 3 at the EAT-10 were considered positive for dysphagia symptoms [9]. Positive patients were contacted by telephone and were offered to undergo a fiberoptic endoscopic evaluation of swallowing (FEES). A sample of age-matched healthy volunteers, recruited for a previous study [10], were used as control group for FEES findings. Both patients and healthy volunteers underwent the same FEES protocol.

### Sleep study

Home sleep cardiorespiratory polygraphy was performed using a portable sleep monitor SOMNOcheck effort (Weinssman medical technology Hamburg D EU). Oro-nasal cannulae and thoraco-abdominal belts with piezo-electrodes respectively recorded airflow and ventilatory efforts; snoring was detected by a digital sound meter included in the portable sleep monitor. Peripheral oxyhemoglobin saturation (SpO2) was recorded by finger pulse oximetry. The percent of recording time spent < 90% of SpO2 was defined as T90. Bedtime and awakening times were set by the technical staff on the portable sleep monitor before placement, according to preferences expressed by patients.

Breathing variables were manually scored in 5 min epochs, according to standardized criteria [11]. Obstructive apneas were defined as ≥ 10 s pauses in breathing with concurrent chest and abdominal movement. Hypopnea was defined as a decrease in airflow ≥ 50%, associated with a fall in pulse oximetry ≥ 4%. The apnea–hypopnea index (AHI) was defined as the average number of episodes of both apnea and hypopnea per hour of sleep [11]. An AHI ≥ 5 was defined as OSA. Mild, moderate, and severe OSA were defined as an AHI of 5–14.9, 15–29.9 and ≥ 30, respectively.

### Clinical data

Clinical data on diseases and symptoms typically associated with OSA were recorded. In particular, the following information was collected:

- *Body mass index* (BMI, kg/m^2^)
- The presence and the total number of the following comorbidities: hypertension, heart failure, diabetes mellitus, thyroid diseases, hypercholesterolemia, hypertriglyceridemia, hiatal hernia, gastric ulcer or gastritis, asthma, rhinitis or rhinosinusitis, and other neurological diseases not included in the exclusion criteria
- The presence of symptoms of gastroesophageal reflux disease as measured by a *GERD-Q questionnaire* score ≥ 8 [12]
- The presence of anxiety and/or depression as reported by the patient
- The presence and the total number of the following OSA symptoms as reported by the patient or the partner: nocturnal motor restlessness, snoring, sudden awakenings, bruxism, nocturnal enuresis, cephalgia, concentration difficulties, drowsy-driving
- The presence of sleepiness as measured by an *Epworth Sleepiness Scale* (ESS) score ≥ 10 [13].

### Dysphagia assessment

Dysphagia symptoms were investigated in all patients with the EAT-10, a 10-item, widely-used questionnaire [8]. A total score is gained ranging from 0 (no problem) to 40 (severe problem). An EAT-10 score ≥ 3 is suggestive of dysphagia. The EAT-10 has been translated and validated in Italian [9].

In symptomatic patients (EAT-10 ≥ 3), FEES was conducted by an otorhinolaryngologist using a XION EF-N flexible endoscope (XION GmbH, Berlin) mounted on an EndoSTROBE camera (XION GmbH, Berlin). FEES was conducted with liquids (3 trials × 5–10–20 cc), semisolids (3 trials × 5–10–20 cc), and solids (2 trials × 8g cracker).

FEES recordings of patients with OSA and healthy volunteers were de-identified and assessed by the same speech and language therapist with 10 years of experience on FEES interpretation, who was blinded to the health status of the subject. The presence and severity of penetration and aspiration, pharyngeal residue, and premature spillage were assessed using the following scales:

- The *Penetration-aspiration scale (PAS)* [14], a validated ordinal scale to assess penetration and aspiration events. The scale ranges from 1 (no penetration-aspiration) to 8 (silent aspiration).
- The *Yale Pharyngeal Residue Severity Rating Scale (YPRSRS)* [15], a validated ordinal scale ranging from 1 (no residue) to 5 (severe residue), to assess the amount of post-swallow residue in the valleculae and the pyriform sinus.
- A 5-point ordinal scale proposed by Langmore [16] to assess the location of the head of the bolus at the onset of swallowing. The score ranges from 0 (head of the bolus is behind the tongue) to 4 (head of the bolus falls into the laryngeal vestibule or is aspirated before the swallow).

Dysphagia pathophysiology was characterized for each patient with OSA according to the videoendoscopic classification proposed by Desuter [17]. The presence of six pathophysiological mechanisms were assessed: protective deficit, posterior oral incontinence, delayed pharyngeal phase, oropharyngeal dyspraxia, propulsion deficit, and resistive issue.

As symptoms of dysphagia may overlap symptoms of laryngopharyngeal reflux (LPR), the presence of LPR was investigated before FEES. Two diagnostic scores were used to detect the presence of LPR: a *Reflux Finding Score (RFS)* ≥ 7 based on videolaryngoscopy [18] and a *Reflux Symptom Index (RSI)* ≥ 13 based on patient-reported symptoms [19].

### Statistical analysis

Inspection of variables’ distribution suggested that the normality assumption was not reasonable. Therefore, data are reported as absolute (relative) frequency and median (IQR). Statistical analysis was performed with the IBM SPSS Statistics 26.0® package for Windows (SPSS Inc, Chicago, IL).

Univariate logistic regression was performed to estimate the degree of association between OSA variables and the presence of dysphagia symptoms. The presence of dysphagia symptoms, according to an EAT-10 score ≥ 3, was the dependent variable. Demographic, clinical, and polysomnographic variables were used as independent variables. Gender, OSA severity (AHI), snoring during the polysomnography, sleepiness (ESS), gastroesophageal reflux (GERD-Q), anxiety/depression, and single comorbidities were used as categorical variables. Age, BMI, total number of comorbidities, total number of OSA symptoms, and polysomnographic parameters were used as continuous variables. Univariate analyses were performed using the chi-squared test for categorical variables and the Mann–Whitney U test for continuous variables. Statistically significant variables at univariate analyses were included in the multivariate logistic regression using forced entry method, which was corrected for age and OSA severity. Measures of association were presented as odd ratio (OR) with 95% confidence intervals (CI 95%). Significance was set at p < 0.05. Patients with missing values were excluded pairwise.

FEES findings were compared between patients with OSA and healthy volunteers using the Mann–Whitney U test. The EAT-10 scores were compared among patients with OSA with and without signs (RFS) and symptoms (RSI) of LPR with the Mann–Whitney U test.

## Results

### Patients

Over 20 months, 1002 patients with a diagnosis of OSA were recruited. Fifty-one patients were excluded because of unreliable or missing data from the polysomnography or missing EAT-10. Thus, 951 patients (666 [70%] males, median age 62 [IQR, 51–71]) were included in the study. The median (IQR) BMI was 28 (25–31) and the AHI was 19 (11–33). OSA was mild in 375 (39%) patients, moderate in 297 (31%) patients, and severe in 279 (29%) patients.

### Dysphagia symptoms: prevalence and associations

Based on the EAT-10, 141 (15%) patients reported symptoms of dysphagia. Patients with dysphagia symptoms had a median EAT-10 of 5 (4–11). Table 1 shows the frequency of scores for each EAT-10 item.

**Table 1:** Frequency of scores on the single items of the EAT-10 in symptomatic patients with OSA

Item	Score				
	0	1	2	3	4
My swallowing problem has caused me to lose weight	104 (73.8%)	13 (9.2%)	10 (7.1%)	12 (8.5%)	2 (1.4%)
My swallowing problem interferes with my ability to go out for meals	103 (73%)	21 (14.9%)	10 (7.1%)	5 (3.5%)	2 (1.4%)
Swallowing liquids takes extra effort	66 (46.8%)	42 (29.8%)	18 (12.8%)	12 (8.5%)	2 (2.1%)
Swallowing solids takes extra effort	57 (40.4%)	43 (30.5%)	18 (12.8%)	16 (11.3%)	7 (5%)
Swallowing pills takes extra effort	53 (37.6%)	44 (31.25)	27 (19.1%)	9 (6.4%)	8 (5.7%)
Swallowing is painful	87 (61.7%)	30 (21.3%)	14 (9.9%)	8 (5.7%)	2 (1.4%)
The pleasure of eating is affected by my swallowing	82 (58.2%)	29 (20.6%)	14 (9.9%)	10 (7.1%)	6 (4.3%)
When I swallow food sticks in my throat	55 (39%)	45 (31.9%)	21 (14.9%)	12 (8.5%)	8 (5.7%)
I cough when I eat	46 (32.6%)	50 (35.5%)	25 (17.7%)	11 (7.8%)	9 (6.4%)
Swallowing is stressful	74 (52.5%)	36 (25.5%)	14 (9.9%)	12 (8.5%)	5 (3.5%)

Each item is scored from 0 (no problem) to 4 (severe problem). Data are reported as frequencies and (prevalence)

Demographic, clinical, and polysomnographic variables of patients with and without symptoms of dysphagia are reported in Tables 2 and 3. Because of missing values, 157 patients were excluded from the multivariate analysis. Anthropometrical and clinical characteristics do not significantly differ between patients that were included and those excluded from the multivariate analysis, except for the variables “other neurological diseases” and “anxiety/depression”, that were more prevalent in the excluded group (Additional file 1: Table S1). The model was based on 794 patients (83.5% of the study sample) (Table 4). Gender (female), sleepiness (ESS), gastroesophageal reflux (GERD-Q), number of OSA symptoms, and anxiety/depression were found to be independently associated with the presence of dysphagia symptoms.

**Table 2:** Comparison between patients with and without symptoms of dysphagia: chi-squared test (categorical variables)

Variable		EAT-10 ≥ 3 (N = 141)	EAT-10 < 3 (N = 810)	p
Gender (F)		68 (48.6%)	217 (27%)	** < 0.001**
AHI	Mild (5–15)	63 (44.7%)	312 (38.5%)	0.421
	Moderate (16–29)	44 (31.2%)	253 (31.2%)	
	Severe (≥ 30)	34 (24.1%)	245 (30.2%)	
ESS ≥ 10		68 (48.6%)	167 (21.2%)	** < 0.001**
Snoring (polysomnography)		93 (66%)	529 (65.2%)	0.986
GERD-Q ≥ 8		62 (32.8%)	127 (16.8%)	** < 0.001**
Anxiety/depression		55 (41%)	150 (19%)	** < 0.001**
Comorbidities	Hypertension	60 (43.8%)	398 (49.9%)	0.189
	Heart failure	18 (17.5%)	85 (10.7%)	0.334
	Diabetes mellitus	30 (22.4%)	94 (11.8%)	**0.001**
	Thyroid diseases	28 (21.2%)	103 (13%)	**0.012**
	Hypercholesterolemia	52 (38%)	286 (36.2%)	0.686
	Hypertriglyceridemia	25 (18.5%)	123 (15.8%)	0.431
	Hiatal hernia	31 (23.1%)	101 (12.8%)	**0.002**
	Gastric ulcer/Gastritis	35 (26.5%)	94 (12%)	** < 0.001**
	Asthma	17 (12.7%)	72 (9.1%)	0.193
	Rhinitis/Rhinosinusitis	24 (18%)	82 (10.5%)	**0.013**
	Neurological diseases^a^	18 (13.4%)	46 (5.9%)	**0.002**

^a^Other than neurological diseases used as exclusion criteria

Significant differences are reported in bold. Data are reported as frequencies and (prevalence)

F, female; AHI, apnea–hypopnea index; ESS, Epworth Sleepiness Scale

**Table 3:** Comparison between patients with and without symptoms of dysphagia: Mann–Whitney test (continuous variables)

Variable	EAT-10 ≥ 3 (N = 141)	EAT-10 < 3 (N = 810)	p
Age	62 (52–72)	62 (52–71)	0.936
BMI	29 (25.5–32.9)	28 (25–31)	0.071
N comorbidities	3 (1–5)	2 (1–3)	** < 0.001**
N symptoms	5 (3–6)	3 (2–4)	** < 0.001**
Apnea i	8 (4–15.7)	9 (4.1–20)	0.075
Hypopnea i	7 (4–13)	7 (4–12.9)	0.480
Average SpO2 (%)	93 (92–94.7)	93 (92–95)	0.342
Nadir SpO2 (%)	81 (75–86)	81 (76–85)	0.608
Sat < 90% (%) T90	7 (1–23)	6 (1–20)	0.904

Significant differences are reported in bold. Data are reported as median (IQR)

BMI, body mass index; SpO2, Peripheral oxyhaemoglobin saturation

**Table 4:** Factors associated with symptoms of dysphagia in patients with OSA (N = 794): a multivariate analysis

	OR (CI 95%)	p
Age	1.01 (0.98–1.02)	0.623
Gender (F)	**2.31 (1.44–3.70)**	**0.001**
AHI severity		0.366
Mild vs moderate/severe	0.70 (0.41–1.19)	0.188
Moderate vs mild/severe	0.75 (0.43–1.32)	0.322
ESS ≥ 10	**2.24 (1.38–3.63)**	**0.001**
GERD-Q ≥ 8	**2.75 (1.70–4.45)**	** < 0.001**
N comorbidities	0.99 (0.83–1.20)	0.985
N symptoms	**1.25 (1.08–1.45)**	**0.002**
Anxiety/Depression	**1.89 (1.17–3.06)**	**0.009**
Diabetes mellitus	1.89 (0.93–3.82)	0.078
Thyroid diseases	0.84 (0.44–1.62)	0.601
Hiatal hernia	0.94 (0.48–1.82)	0.848
Gastric ulcer/Gastritis	1.67 (0.92–3.05)	0.093
Rhinitis/Rhinosinusitis	1.16 (0.58–2.35)	0.675
Neurological diseases^a^	1.52 (0.67–3.48)	0.321

^a^Other than neurological diseases used as exclusion criteria

Significant associations are reported in bold. Data are reported as OR (CI 95%). The model correctly classified 87% of the patients

F, female; AHI, apnea–hypopnea index; ESS, Epworth Sleepiness Scale

### FEES findings in symptomatic patients

Thirty-five patients with OSA (15 [44%] males, median age 61 [IQR, 48–70] and symptoms of dysphagia (EAT-10 ≥ 3) accepted to undergo an instrumental assessment of swallowing by FEES. The median (IQR) BMI was 31 (26–34) and the AHI was 13 (8–24). OSA was mild in 21 (60%) patients, moderate in 7 (20%) patients, and severe in 7 (20%) patients. Main reasons for refusing FEES were: other comorbidities requiring frequent medical visits or surgery (46%), inability to contact the patient by telephone (39%), instrumental assessment of swallowing already performed (7%), difficulties in reaching the hospital (4%), and fear of FEES (4%). Patients who accepted FEES did not significantly differ from those who did not perform FEES for the total and single items’ EAT-10 score, AHI, ESS, age, gender, BMI, anxiety/depression, number of symptoms, and number of comorbidities (Mann–Whitney U test p > 0.05) (Additional file 1: Table S2).

**Fig. 1:**
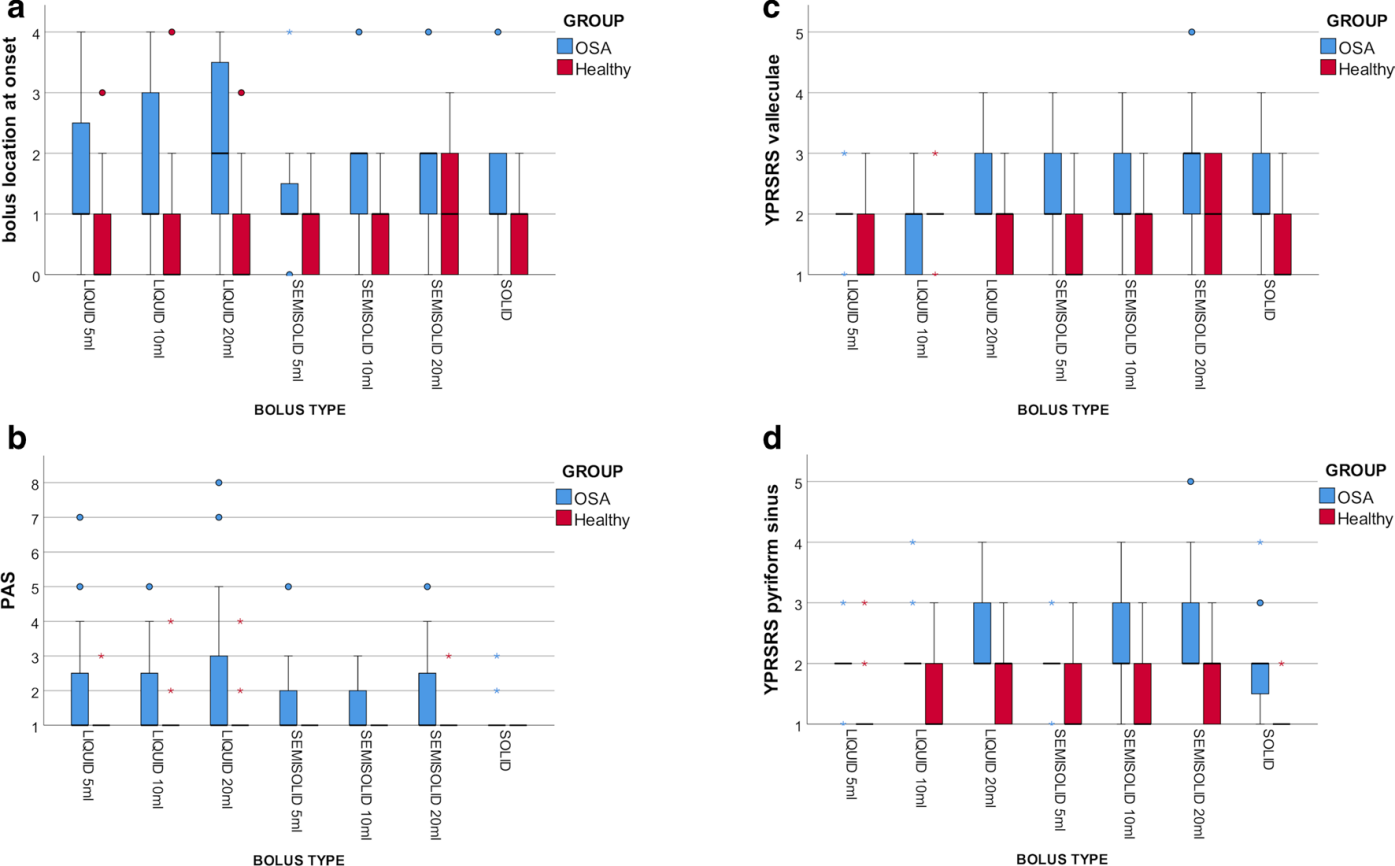
Comparison of FEES findings between symptomatic patients with OSA and healthy subjects. In the box plots, vertical solid lines (whiskers) show lower and upper scores of the different ordinal scales. Box stretches from lower hinge (25th percentile) to upper hinge (75th percentile). Median is shown as line across each box. Outsiders are represented by dots, extreme values are represented by asterisks. **a** Comparison of the bolus location at swallow onset, scored with the 5-point ordinal scale by Langmore et al^16^, between patients with OSA and healthy subjects (Mann–Whitney U test p = 0.077 for solids, p < 0.05 for all other bolus types). **b** Comparison of penetration and aspiration events, scored with the PAS, between patients with OSA and healthy subjects (Mann–Whitney U test p < 0.05 for all bolus types). **c** Comparison of the pharyngeal residue in the valleculae, scored with YPRSRS, between patients with OSA and healthy subjects (Mann–Whitney U test p < 0.05 for all bolus types). **d** Comparison of t the pharyngeal residue in the pyriform sinus, scored with YPRSRS, between patients with OSA and healthy subjects (Mann–Whitney U test p < 0.05 for all bolus types). PAS, Penetration-aspiration scale; YPRSRS, Yale Pharyngeal Residue Severity Rating Scale

FEES detected one or more clinically significant signs of dysphagia in 34/35 symptomatic patients with OSA. FEES findings of the 35 symptomatic patients with OSA were compared with those of 27 age-matched healthy subjects (11 [41%] males, median age 58 [IQR, 41-70]). Regardless of the bolus type, patients with OSA showed significantly (Mann–Whitney U test p < 0.05) lower location of the bolus at swallow onset (except for solids), greater pharyngeal residue both in the valleculae and the pyriform sinus, and higher frequency and severity of penetration and aspiration events than healthy subjects (Fig. 1). The study of pathophysiological mechanisms of dysphagia revealed that 22 (63%) patients had delayed pharyngeal response, 14 (40%) patients poor oral control, 13 (37%) patients propulsive deficit, 3 (9%) patients protective deficit, and 2 (6%) patients oropharyngeal dyspraxia.

**Fig. 2:**
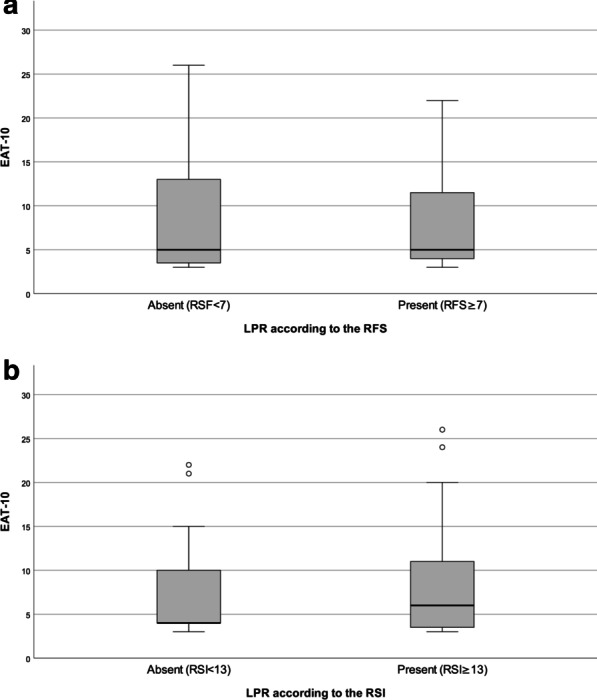
Comparison of the EAT-10 score between patients with and without LRP. In the box plots, vertical solid lines (whiskers) show lower and upper EAT-10 scores. Box stretches from lower hinge (25th percentile) to upper hinge (75th percentile). Median is shown as line across each box. Outsiders are represented by dots. **a** Comparison of the EAT-10 score between patients without LPR (n = 20, EAT-10 median 5, IQR 3.3–14) and patients with LPR (n = 15, EAT-10 median 5, IQR 3.8–13.3) according to the RFS (Mann–Whitney U test p = 0.934). **b** Comparison of the EAT-10 score between patients without LPR (n = 12, EAT-10 median 4, IQR 4–15) and patients with LPR (n = 23, EAT-10 median 6, IQR 3–11) according to the RSI (Mann–Whitney U test p = 0.717)

Concerning the LPR, among symptomatic patients who underwent FEES, 15 (43%) patients had a RSF ≥ 7, while 23 (66%) patients a RSI ≥ 13, suggestive of LPR. EAT-10 total and single-item scores did not significantly differ between patients with and without LPR, regardless of the diagnostic criteria (Fig. 2).

## Discussion

The main findings of the present study can be summarized as follows: (1) in a cohort of 951 patients with OSA, 15% of patients reported symptoms of dysphagia; (2) higher OSA symptoms, greater sleepiness, anxiety/depression, concomitant gastroesophageal reflux, and female gender were independently associated with dysphagia symptoms; (3) FEES confirmed the presence of dysphagia in almost all symptomatic patients with OSA, which was mainly related to delayed pharyngeal reflex, poor oral control, and bolus propulsion deficit.

Based on an EAT-10 score ≥ 3, 15% of patients with OSA presented with symptoms of dysphagia. The prevalence of dysphagia symptoms in the general population was 8%, using the same questionnaire and cut-off [20]. The present results suggest that a diagnosis of OSA may nearly doubles the prevalence of dysphagia symptoms. The literature suggests that dysphagia in patients with OSA may be the result of three main mechanisms. Firstly, it was hypothesized that dysphagia may derive from a sensory alteration of the pharynx secondary to low-frequency vibrations, intermittent hypoxia, and inflammatory changes [3, 21]. Secondly, a motor component has been described. In fact, patients with OSA exhibit altered appearance and increased fatigability of orofacial and pharyngeal musculature, as well as altered composition of muscle fibres [4, 22]. Finally, altered swallowing-breathing integration has been reported [3, 5, 23].

The prevalence of dysphagia symptoms of our cohort confirms the results of the Japanese study [7], which reported a prevalence of 16%. Although with different questionnaires, both studies used multiple items-patient-reported tools to investigate dysphagia symptoms. The assessment of dysphagia symptoms should not be limited to one single open question on swallowing function, due to its insufficient diagnostic performance [24]. It appears advisable to integrate the assessment of patients with OSA in clinical practice with a validated, rapid, and dysphagia-specific tool, such as the EAT-10, that can help the identification of candidates for an in-depth swallowing assessment, preventing dysphagia-related complications. Early identification of dysphagia would allow reducing dysphagia-related pulmonary, nutritional, and psychosocial consequences [25–27]. Among these, preventing the development of aspiration pneumonia is particularly important in this population, being OSA itself is a risk factor for pneumonia [28, 29].

The present study identified several independent factors associated with dysphagia symptoms, which may additionally guide clinicians in the selection of patients at higher risk of dysphagia. The former includes female gender, OSA symptomatology, anxiety/depression, and gastroesophageal reflux. The multivariate analysis was performed including almost 85% of the overall sample because of missing values. Indeed, excluded patients showed comparable anthropometrical and clinical characteristics compared to the rest of the population, except for a higher prevalence of anxiety and depression and neurological conditions without known association with dysphagia. However, anxiety and depression were significant predictors of dysphagia in the multivariate analysis, and the neurological diseases that may act as confounder in the diagnosis of dysphagia were excluded from the study, thus, we believe that the risk of selection bias was low and did not interfere with the validity of our results.

The association with the female gender was unexpected. OSA is more prevalent in males and both the pathophysiology and the evolutionary anatomic changes pose male patients at higher risk of more severe disease in respect to women [29–32]. To the best of our knowledge, the mechanisms and pathophysiology of sleep apnea are not gender-related, and females with OSA should not be at higher risk of dysphagia. Furthermore, to date, a gender effect on swallowing function was not recognized [33]. One study reported a similar trend for higher prevalence of dysphagia in OSA females patients [34]. The authors hypothesized that the observation was influenced by confounding factors, such as aging. The present study seems not to support this hypothesis. In the absence of clear evidence, future studies should shed light on the association between gender and dysphagia in OSA.

Interestingly, age was not associated with dysphagia symptoms in our sample. Previous studies reported conflicting evidence, favoring [8, 34] and in contrast to our result [35]. It is known that dysphagia prevalence increases with aging [36]. The lack of association between age and dysphagia symptoms in the present study, might suggest that dysphagia arises as a consequence of OSA itself and not from the normal aging process.

Analogously, no association was found between OSA severity and dysphagia symptoms, in accordance with previous reports [8, 34, 35]. Conversely, we found that dysphagia symptoms were more frequently reported by patients with a higher burden of OSA-related symptoms and greater daytime sleepiness. Excessive daytime sleepiness does not seem to be associated with OSA severity as measured by AHI [37]. The main hypotheses are that excessive daily sleepiness might be either the effect of REM-dependent OSA or the result of lower oxygenation during sleep [38–40]. As repetitive nocturnal hypoxemia has been identified in OSA patients with impaired swallowing [41], it may be hypothesised that it could represent the common underlying mechanism of dysphagia and excessive daytime sleepiness in OSA. However, we did not find an association between dysphagia symptoms and SpO2 indices. Future studies should provide a more in-depth insight on the association between dysphagia and daily sleepiness in OSAS, investigating other polysomnographic indices and objective measures of dysphagia.

Affective symptoms are common in patients with dysphagia [42]. The relation between dysphagia and anxiety and depression may be bidirectional. On one hand, dysphagia may arise as a consequence of the effects of medications on swallowing function [43]. Moreover, anxiety and depression might influence the perception of swallowing, with patients being more prone to report dysphagia symptoms. On the other hand, dysphagia may increase the prevalence of affective symptoms by altering eating habits and limiting social participation [42].

Gastroesophageal reflux is known to be associated with OSA [44]. We found a significant association between symptoms of gastroesophageal reflux and symptoms of dysphagia in OSA. Dysphagia and gastroesophageal reflux disease have been reported to be concomitant conditions in other populations [45–47]. In patients with gastroesophageal reflux, dysphagia was associated with cricopharyngeal incoordination [48] and delayed airway closure [49], potentially related to reflux-induced sensory impairment.

It could be speculated that symptoms of reflux may simulate symptoms of dysphagia recorded by the EAT-10. In patients who underwent FEES, both objective signs and subjective symptoms of LPR were investigated. No significant differences were found in the EAT-10 scores between patients with and without LPR, regardless of the scale used to assess it. Thus, this result seems to confirm that, in the study sample, the EAT-10 recorded symptoms of dysphagia and not symptoms of reflux, corroborating the finding by Caparroz on the fact that dysphagia and LPR, although may be concomitant, are not associated conditions in OSA [34]. However, as this analysis was based on the small sample size of patients who underwent FEES, the potential for an influence of reflux (either gastroesophageal or laryngopharyngeal) on the EAT-10 scores should be recognized and the prevalence data should be interpreted cautiously.

FEES confirmed the presence of dysphagia in 97% of the symptomatic patients who accepted the examination. Other studies instrumentally assessing swallowing function in OSA reported a lower frequency of signs of dysphagia, ranging from 20 to 77% [6]. Previous studies included both symptomatic and asymptomatic patients. Our results may suggest that the selection of OSA candidates to swallowing assessment based on a standardized patient-reported tool could increase the appropriateness of the instrumental examination. However, the high rate of FEES refusal in our study may be responsible for the higher prevalence of instrumentally documented dysphagia, by selecting more symptomatic or concerned patients. Therefore, future studies on larger samples should confirm the present data.

At FEES, patients with OSA exhibited impaired swallowing safety and reduced swallowing efficiency. The main pathophysiological mechanism of dysphagia was a delayed pharyngeal response, aligned with previous findings [35, 50, 51]. Other motor deficits, such as reduced propulsion of the bolus, were also reported but in a smaller portion of the sample. It confirms that both sensory and motor changes associated with OSA impairs swallowing function, but the sensory component seems to be predominant.

The study has some limitations. First, we investigated the prevalence of dysphagia symptoms and of dysphagia as objectively diagnosed by instrumental assessment. This choice was related to the feasibility of analyzing dysphagia on a large sample (N = 951). Indeed, the EAT-10 represents an easy-to-use and rapid tool, whereas instrumental swallowing assessments, either with FEES or videofluoroscopy, are minimally invasive procedures. Nevertheless, using a patient-reported outcome might have underestimated the real prevalence of dysphagia because of poor awareness of patients. Conversely, as the study was conducted in an academic institution, the prevalence of dysphagia symptoms in OSA may have been overestimated due to a referral bias of more complicated OSA patients. Second, as previously stated in the discussion, although patients who refused and patients who accepted FEES did not significantly differ for main factors influencing OSA and dysphagia, the large number of symptomatic patients with OSA who refused to undergo the FEES might have led to a selection bias. Finally, being an observational study on a large sample size, standard polysomnographic indices routinely used in clinical practice were investigated. The present study failed to detect an association between polysomnographic indices and dysphagia symptoms in patients with OSA. However, measuring more specific arousal indices and providing a full characterization of obstructive hypopneas could lead to different results. Future studies should expand the investigation including other polysomnographic indices.

## Conclusions

One-sixth of patients with OSA exhibit symptoms of dysphagia. Dysphagia is independently associated with female gender, OSA symptoms, excessive daily sleepiness, anxiety/depression, and gastroesophageal reflux. Including a screening of dysphagia for patients with OSA in clinical practice appears advisable. The EAT-10 seems to be a sensitive tool to guide the selection of patients at high risk of dysphagia.

## Supplementary Information

**Additional file 1: Table S1.** Comparison between patients included and excluded (missing data) in the multivariate analysis. **Table S2.** Comparison between symptomatic patients who accepted and who refused the fiberoptic endoscopic evaluation of swallowing.

## Data Availability

The datasets used and analyzed during the current study are available from the corresponding author on reasonable request.

## Abbreviations

AHI: Apnea–hypopnea index
BMI: Body mass index
EAT-10: Eating-Assessment Tool
ESS: Epworth Sleepiness Scale
IQR: Interquartile range
LPR: Laryngopharyngeal reflux
FEES: Fiberoptic endoscopic evaluation of swallowing
OSA: Obstructive sleep apnea
PAS: Penetration-aspiration scale
RFS: Reflux Finding score
RSI: Reflux Symptom Index
SpO2: Peripheral oxyhaemoglobin saturation
YPRSRS: Yale Pharyngeal Residue Severity Rating Scale
