# Supplementary material for: Dysphagia symptoms in obstructive sleep apnea: prevalence and clinical correlates
Source: Respir Res. 2021 Apr 21;22:117. doi: 10.1186/s12931-021-01702-2 (PMC8061009; doi:10.1186/s12931-021-01702-2)
Supplement: Supplementary file 1 — Additional file 1: Table S1. Comparison between patients included and excluded (missing data) in the multivariate analysis. Table S2. Comparison between symptomatic patients who accepted and who refused the fiberoptic endoscopic evaluation of swallowing. [file 12931_2021_1702_MOESM1_ESM.docx]

**SUPPLEMENTARY MATERIAL**

**Table 1S. Comparison between patients included and excluded (missing data) in the multivariate analysis**

| **Variable** | **Included**  **(n=794)** | **Excluded**  **(n=157)** | **p** |
| --- | --- | --- | --- |
| *EAT-10 ≥3* | 116 (14.6%) | 25 (15.9%) | 0.672 |
| *Age* | 62 (51.5-71) | 63 (53-72) | 0.489 |
| *Gender (F)* | 234 (29.5%) | 51 (32.5%) | 0.315 |
| *BMI* | 28 (25-31) | 28 (25-32) | 0.681 |
| *AHI mild (5-12)* | 307 (38.7%) | 68 (43.3%) | 0.494 |
| *moderate (16-29)* | 249 (31.4%) | 48 (30.6%) |  |
| *Severe (>=30)* | 238 (30%) | 41 (26.1%) |  |
| *AHI (continue)* | 19 (11-33) | 17 (10-30) | 0.127 |
| *Apnea i* | 9 (4.3-20) | 7.9 (4-18) | 0.227 |
| *Hypopnea i* | 7 (4-13) | 7 (4-12) | 0.407 |
| *Average SpO2 (%)* | 93 (92-95) | 93 (92-95) | 0.589 |
| *Nadir SpO2 (%)* | 81 (76-85) | 81 (74-85) | 0.427 |
| *Sat <90% (%) T90* | 6 (1-21) | 6 (2-20) | 0.825 |
| *Snoring (polysomnography)* | 519 (76.5%) | 103 (65.6%) | 0.057 |
| *ESS ≥10* | 203 (25.6%) | 32 (20.4%) | 0.712 |
| *GERD-Q ≥8* | 161 (20.3%) | 28 (17.8%) | 0.051 |
| *Anxiety/depression* | 163 (20.5%) | 41 (26.1%) | **0.005** |
| *N symptoms* | 3 (2-5) | 3 (2-4) | 0.329 |
| *N comorbidities* | 2 (1-4) | 3 (1-4) | 0.223 |
| *Hypertension* | 383 (48.4%) | 75 (47.8%) | 0.375 |
| *Heart failure* | 83 (10.5%) | 20 (12.7%) | 0.159 |
| *Diabetes mellitus* | 97 (12.2%) | 27 (17.2%) | 0.110 |
| *Thyroid diseases* | 112 (14.1%) | 19 (12.1%) | 0.905 |
| *Hypercholesterolemia* | 281 (35.7%) | 57 (36.3%) | 0.227 |
| *Hypertriglyceridemia* | 120 (15.4%) | 28 (17.8%) | 0.084 |
| *Hiatal hernia* | 109 (13.8%) | 23 (14.6%) | 0.196 |
| *Gastric ulcer/Gastritis* | 109 (13.7%) | 19 (15.4%) | 0.609 |
| *Asthma* | 71 (9%) | 18 (12.1%) | 0.086 |
| *Rhinitis/Rhinosinusitis* | 89 (11.0%) | 19 (12.1%) | 0.089 |
| *Neurological diseases*^a^ | 48 (6%) | 16 (10.2%) | **0.003** |
|  |  |  |  |

^a^ Other than neurological diseases used as exclusion criteria

Notes. Data are reported as median (IQR) for continuous variables and as n (%) for categorical variables. P-values refers to the Mann-Whitney U test for continuous variables and to the Chi-squared test for categorical variables. Significant p-values are reported in bold.

Legend. EAT-10, Eating Assessment Tool; F, female; BMI, body mass index; AHI, apnea-hypopnea index; ESS, Epworth Sleepiness Scale

**Table 2S. Comparison between symptomatic patients who accepted and who refused the fiberoptic endoscopic evaluation of swallowing**

| **Variable** | **Accepted FEES**  **N=35** | **Refused FEES**  **N=106** | **p** |
| --- | --- | --- | --- |
| *Age* | 61 (49.8-70) | 62 (52.8-72.3) | 0.349 |
| *Gender (F)* | 20 (57%) | 48 (45%) | 0.475 |
| *BMI* | 30.1 (25.9-34) | 28.6 (25-30.6) | 0.182 |
| *AHI* | 12.5 (8-24.1) | 16.3 (10.2-31.2) | 0.160 |
| *ESS* | 9 (6-14.3) | 9 (6-13) | 0.518 |
| *N comorbidities* | 4 (2-5) | 3 (1-4.3) | 0.325 |
| *N symptoms* | 5 (3.8-6) | 4 (3-6) | 0.061 |
| *Anxiety/depression* | 14 (40%) | 41 (39%) | 0.853 |
| *EAT-10 total score* | 5.5 (3.8-12.8) | 5 (4-10.3) | 0.931 |
| *EAT-10 item 1* | 0 (0-0.3) | 0 (0-1) | 0.537 |
| *EAT-10 item 2* | 0 (0-0) | 0 (0-1) | 0.356 |
| *EAT-10 item 3* | 0.5 (0-1) | 1 (0-2) | 0.407 |
| *EAT-10 item 4* | 1 (0-1.3) | 1 (0-2) | 0.653 |
| *EAT-10 item 5* | 1 (0-2) | 1 (0-2) | 0.447 |
| *EAT-10 item 6* | 0 (0-1) | 0 (0-1) | 0.426 |
| *EAT-10 item 7* | 0 (0-1) | 0 (0-1) | 0.245 |
| *EAT-10 item 8* | 1 (0-2) | 1 (0-2) | 0.492 |
| *EAT-10 item 9* | 1 (0-2) | 1 (0-2) | 0.895 |
| *EAT-10 item 10* | 1 (0-2) | 0 (0-1) | 0.407 |

Notes. Data are reported as median (IQR) for continuous variables and as n (%) for categorical variables. P-values refers to the Mann-Whitney U test for continuous variables and to the Chi-squared test for categorical variables.

Legend. F, female; AHI, apnea-hypopnea index; BMI, Body Mass Index; ESS, Epworth Sleepiness Scale
